# Reported antibiotic use among patients in the multicenter ANDEMIA infectious diseases surveillance study in sub-saharan Africa

**DOI:** 10.1186/s13756-024-01365-w

**Published:** 2024-01-25

**Authors:** Imke Wieters, Siobhan Johnstone, Sheila Makiala-Mandanda, Armel Poda, Chantal Akoua-Koffi, Muna Abu Sin, Tim Eckmanns, Valentina Galeone, Firmin Nongodo Kaboré, François Kahwata, Fabian H. Leendertz, Benoit Mputu, Abdoul-Salam Ouedraogo, Nicola Page, Susanne B. Schink, Fidèle Sounan Touré, Adjaratou Traoré, Marietjie Venter, Ann Christin Vietor, Grit Schubert, Sara Tomczyk

**Affiliations:** 1 Institute of Tropical Medicine and International Health, Charité– Universitätsmedizin Berlin, Campus Virchow-Klinikum, Augustenburger Platz 1, 13353 Berlin, Germany; 2 Robert Koch-Institute, Nordufer 20, 13353 Berlin, Germany; 3 Center for Enteric Diseases, National Health Laboratory Service, National Institute for Communicable Diseases, 1 Modderfontein Road, Sandringham, Johannesburg, 2131 South Africa; 4 Cliniques Universitaires de Kinshasa, Université de Kinshasa, Kinshasa, Democratic Republic of the Congo; 5 Institut National de Recherche Biomédicale, Kinshasa, Democratic Republic of the Congo; 6 Centre Hospitalier Universitaire Sourô Sanou de Bobo-Dioulasso, Bobo-Dioulasso, Burkina Faso; 7 Centre Hospitalier Universitaire Bouaké, Bouaké, Ivory Coast; 8 Université Alassane Ouattara de Bouaké, Bouaké, Ivory Coast; 9 Centre Muraz, Bobo-Dioulasso, Burkina Faso; 10 Helmholtz Institute for One Health, Fleischmannstraße 42, 17489 Greifswald, Germany; 11 School of Health Systems and Public Health, Faculty of Health Sciences, University of Pretoria, 31 Bophelo Rd, Prinshof 349-Jr, Pretoria, 0084 South Africa; 12 Department of Medical Virology, University of Pretoria, Pretoria, South Africa

**Keywords:** Antimicrobial resistance, Antibiotic use, WHO AWaRe classification, Low- and middle-income countries, Sub-saharan Africa

## Abstract

**Background:**

Exposure to antibiotics has been shown to be one of the drivers of antimicrobial resistance (AMR) and is critical to address when planning and implementing strategies for combatting AMR. However, data on antibiotic use in sub-Saharan Africa are still limited. Using hospital-based surveillance data from the African Network for Improved Diagnostics, Epidemiology and Management of Common Infectious Agents (ANDEMIA), we assessed self-reported antibiotic use in multiple sub-Saharan African countries.

**Methods:**

ANDEMIA included 12 urban and rural health facilities in Côte d’Ivoire, Burkina Faso, Democratic Republic of the Congo, and Republic of South Africa. Patients with acute respiratory infection (RTI), acute gastrointestinal infection (GI) and acute febrile disease of unknown cause (AFDUC) were routinely enrolled, and clinical, demographic, socio-economic and behavioral data were collected using standardized questionnaires. An analysis of ANDEMIA data from February 2018 to May 2022 was conducted. Reported antibiotic use in the ten days prior to study enrolment were described by substance and by the WHO AWaRe classification (“Access”, “Watch”, “Reserve”, and “Not recommended” antibiotics). Frequency of antibiotic use was stratified by location, disease syndrome and individual patient factors.

**Results:**

Among 19,700 ANDEMIA patients, 7,258 (36.8%) reported antibiotic use. A total of 9,695 antibiotics were reported, including 54.7% (*n* = 5,299) from the WHO Access antibiotic group and 44.7% (*n* = 4,330) from the WHO Watch antibiotic group. The Watch antibiotic ceftriaxone was the most commonly reported antibiotic (*n* = 3,071, 31.7%). Watch antibiotic use ranged from 17.4% (56/322) among RTI patients in Côte d’Ivoire urban facilities to 73.7% (630/855) among AFDUC patients in Burkina Faso urban facilities. Reported antibiotic use included WHO Not recommended antibiotics but no Reserve antibiotics.

**Conclusions:**

Reported antibiotic use data from this multicenter study in sub-Saharan Africa revealed a high proportion of WHO Watch antibiotics. Differences in Watch antibiotic use were found by disease syndrome, country and health facility location, which calls for a more differentiated approach to antibiotic use interventions including further evaluation of accessibility and affordability of patient treatment.

**Supplementary Information:**

The online version contains supplementary material available at 10.1186/s13756-024-01365-w.

## Background

Antimicrobial resistance (AMR) poses a major global health threat. It was estimated that bacterial AMR was associated with 4.95 million deaths worldwide in 2019 [1]. It was highest in western sub-Saharan Africa, the estimated burden of people dying due to infections with resistant pathogens was 23.7 per 100,000 (95% Confidence Interval [CI]: 18.2–30.7 per 100,000), which is 7.3 per 100,000 above the global mean [1].

Exposure to antibiotics has been shown to be a driver of AMR in certain settings [2] and is therefore, critical to address when planning and implementing strategies for combatting AMR [1, 3, 4].

A systematic assessment of antibiotic consumption per capita across 76 countries from 2000 to 2015 by Klein et al. revealed an alarming overall increase of 39% in the antibiotic consumption rate over the study period (11.3 to 15.7 DDDs per 1,000 inhabitants per day) [5]. The total per capita consumption of antibiotics was considerably lower in low- and middle-income countries (LMICs) compared to high-income countries (HICs), although there was a large relative increase in antibiotic consumption over the given period in LMICs [5], highlighting the importance of intensified research on antibiotic use across different settings.

To monitor antibiotic use and guide the implementation of antimicrobial stewardship policies, the World Health Organization (WHO) introduced the AWaRe classification framework in 2017, which groups antibiotics into four categories: “Access” (i.e. essential first- or second-line empiric treatment), “Watch” (i.e. antibiotics with a higher risk for selection of resistance and for specific indications only), “Reserve” (i.e. last resort antibiotics for confirmed or highly suspected infections due to multi-drug resistant organisms) and “Not Recommended” antibiotics (i.e. antibiotics or combinations that are not recommended) [6, 7]. In the “Adopt AWaRe” campaign, WHO proposed a target that by 2023 at least 60% of national antibiotic consumption should come from the Access antibiotics group [8].

Another study by Klein et al., applying the AWaRe classification, demonstrated global per capita increases in the consumption of Watch group antibiotics by 90.9% and Access group antibiotics by 26.2% from 2000 to 2015 [9]. The increase in Watch antibiotic consumption was greater in LMICs (165%) compared to HICs (27.9%). Furthermore, the proportion of countries where Access antibiotics accounted for at least 60% of the total antibiotic consumption decreased from 76% (50/66) of countries in 2000 to 55% (42/76) of countries in 2015. In the first study, Klein et al. had found that the consumption rate of cephalosporins, macrolides and quinolones increased among LMICs. The consumption of broad-spectrum penicillins, carbapenems and polymyxins had increased across all country income groups, with a pronounced increase in upper middle-income countries [5].

However, in these global assessments of antibiotic consumption, LMICs, particularly those in sub-Saharan Africa, were largely excluded due to lack of data. In many LMICs, there is a high burden of infectious diseases and infections caused by antimicrobial resistant organisms [1, 10]. Yet, access to diagnostic testing and affordable antibiotic treatment options often remains limited in these settings [11–14]. Other health system factors such as lack of health care infrastructure, available personnel or substandard medications may also further complicate the situation of antimicrobial use across low-resource settings. These factors may lead to empiric or self-medication with inappropriate or inadequate antibiotics and/or incorrect dosing, although a holistic approach is needed when considering the complex range of factors affecting antimicrobial use in such settings [15–21].

Generating antibiotic consumption or use data is, thus, of utmost importance to evaluate antimicrobial stewardship efforts especially in low-resource settings. To address this gap, we aimed to describe reported initial antibiotic use among patients with respiratory infection (RTI), gastrointestinal infection (GI) and acute fever of unknown origin (AFDUC) in a multicenter hospital-based surveillance study in sub-Sahara Africa. We sought to assess self-reported antibiotic use according to the WHO AWaRe classification and by location and patient factors in order to provide further insights into antibiotic use in these settings where such data are urgently needed.

## Methods

### Study design and population

A descriptive analysis of data from the ANDEMIA (African Network for Improved Diagnostics, Epidemiology and Management of Common Infectious Agents) infectious disease surveillance network was conducted using data from 1st February 2018 to 26th May 2022. ANDEMIA is a transnational sentinel surveillance network including 12 urban and rural sentinel health care facilities in Côte d’Ivoire (CIV), Burkina Faso (BF), Democratic Republic of Congo (DRC) and Republic of South Africa (RSA). Country and study site characteristics are provided in the supplementary text (see Additional File 1 and 2) [22].

### Data collection

Since 2018, the ANDEMIA surveillance network has been collecting clinical, epidemiological, and laboratory data on patients with acute febrile disease of unknown cause (AFDUC), community-acquired gastrointestinal infection (GI), and respiratory tract infection (RTI). The network aims to expand the understanding of etiologies causing these syndromes, including AMR, and to build capacity for infection prevention and control [22]. Data collection procedures including case definitions were previously described by Schubert et al. (see Additional File 1 and 2) [22]. In summary, efforts were made to enroll patients in the 24 h following presentation to the health care facility. Following informed consent and study enrolment, trained staff completed the case investigation form together with the participants or caregivers for minors prior to collection of study specimens. This questionnaire comprised questions on patient demographics, current symptoms, medical history, past and current hospitalization, medication as well as data on individual occupation, socio-economics, housing, water, sanitation, and hygiene (WaSH) facilities, and animal exposures. Antibiotic treatment, including name and date of last dose, taken in the last ten days prior to study inclusion was recorded (see antibiotic use questions in Case Investigation Form in Additional file 3) and therefore subsumes (1) patient self-medication with antibiotics before presentation to the health facility in the last ten days prior to enrolment, (2) prescribed antibiotic use before presentation to the health facility in the last ten days prior to enrolment and, (3) antibiotic use in the enrolling health facility before enrolment if the study samples had not yet been collected. All data were entered into an ANDEMIA study customized database by trained data clerks. Regular plausibility checks, data management reports and validations as well as regular trainings were carried out to improve data quality as described in the published ANDEMIA study protocol [22].

### Data analysis

For this study, selected questionnaire data were extracted and further cleaned. All analyses were performed using Stata (StataCorp. 2021. Stata Statistical Software: Release 17. College Station, TX: StataCorp LLC). Antibiotic use was defined as self-reported use of one or more antibiotics in the ten days prior to study enrolment, including those with a valid date of the last dose recorded on the case investigation form. Antibiotics were analysed by total reported antibiotics and by patients who received one or more antibiotics. Antibiotics that were erroneously reported elsewhere on the case investigation form (e.g. under “other medication”) and antibiotics for which no date was given were summarised separately. Reported antibiotics were grouped according to the WHO AWaRe classification: “Access”, “Watch”, “Reserve”, and “Not Recommended” antibiotics [6]. Guided by the essential medicine lists of the ANDEMIA countries [23–26], the route of administration of reported antibiotics was coded according to expert clinical opinion into the following strata: “parenteral only” (i.e. only parenteral formulation exists), “oral only”, and “both (parenteral and oral) or other possible routes of administration”. If the essential medicine lists in the countries differed, the reported antibiotic was coded in the third strata “both (parenteral and oral) or other possible routes of administration”. The coding frameworks for the antibiotic formulation and WHO AWaRe criteria are provided in the supplementary text (see Additional File 4). Other variables such as climatic region, dry/wet zone, enrolment before or during the COVID-19 pandemic were also created. The start date of the pandemic was set to 11 March 2020 as it was officially declared by the WHO. Biometric measures were calculated according to WHO recommendations including the Z-score with percentiles for 0–5 years [27] and 5–19 years [28] and the Body Mass Index (BMI) classification for adults [29]. These biometric measurement results were combined into a single variable for weight classification ranging from underweight, normal weight, overweight to obesity (see Additional file 5).

We first described the overall ANDEMIA study population by country and patient factors. The absolute and relative frequencies of antibiotic use among enrolled patients were then analysed by country, urban or rural health care facility, patient infectious syndrome, age group, sex, level of education, employment (if minor, of the respective parent or legal caregiver), residence (reported village or city), the time of study enrolment (before or during the COVID-19 pandemic), and clinical factors, weight classification, reported co-morbidities, other medications (antimalarials, antiretrovirals, anti-tuberculosis agents as well as other medicines), the onset of symptoms and current hospitalization. The reported antibiotics were classified by antibiotic agent and according to the WHO AWaRe classification and analysed by health facility location, syndrome complex and patient factors. The ratio of Access-to-Watch antibiotics was calculated in the different patient groups. The number of antibiotics per patient and routes of antibiotic administration were also summarised overall and by health facility location and patient factors. A sensitivity analysis restricting antibiotic use reported only on the same day of enrolment (versus in the full period of ten days prior to study enrolment) was also conducted.

### Ethics

The ANDEMIA network surveillance study adheres to the respective national legislation and ethical standards as well as the Declaration of Helsinki. In all participating countries including Germany, institutional ethics approval was obtained (see the Declaration section below for details). ANDEMIA study objectives were explained, either verbally or in writing, to all study participants (in the case of minors, to parents or legal caregivers) by trained surveillance officers and, prior to obtaining written informed consent. Patient data were pseudonymized upon entry into the database and only accessible to selected trained study staff [22].

## Results

### Overall characteristics of ANDEMIA study participants

Between 1 February 2018, and 26 May 2022, 19,700 patients were enrolled in the ANDEMIA study, including 5,529 (28.1%) from CIV; 4,802 (24.4%) from BF; 5,937 (30.1%) from DRC and 3,432 (17.4%) from RSA. Across all countries, 36.6% (*n* = 7,203) of patients presented with AFDUC, 33.9% (*n* = 6,676) with RTI, 25.8% (*n* = 5,085) with GI and 3.7% (*n* = 736) with both RTI and GI. Approximately half of enrolled patients were children under the age of five and 48.6% were female. More were recruited in health facilities from urban areas (*n* = 12,527, 63.6%) than from rural areas (*n* = 7,173, 36.4%), although 44.0% (8,624/19,617) of overall enrolled patients with data on place of residence reported living in a village (this included 2,413 patients recruited from urban health facilities). A greater proportion of adult patients or legal guardians of minors reported no education in CIV (*n* = 2,987, 54.0%) and BF (*n* = 3,166, 66.4%) compared to DRC (*n* = 876, 14.8%) and RSA (*n* = 63, 1.9%). During the study period, approximately half of patients were enrolled before the COVID-19 pandemic (*n* = 10,604, 53.8%). Although data showed a hospitalization rate of 98.6% (11,009/11,186), data on hospitalization were largely incomplete for CIV (51.8%; 2,861 missing entries) DRC (47.3%; 2,809 missing entries) and BF (29.3%, 1,408 missing entries). A summary table of patient characteristics by country is provided in the supplementary text (see Additional File 6).

### Characteristics of study participants reporting antibiotic use

Among 19,700 ANDEMIA patients, 7,258 (36.8%) reported antibiotic use in the ten days prior to study enrolment. During the study period, slightly more than half of patients with self-reported antibiotic use were enrolled before the COVID-19 pandemic (*n* = 4,319, 59.5%). In CIV, BF, and DRC, more than 80% of patients with reported antibiotic use presented to urban health facilities, in contrast to RSA where the presentation to urban and rural health facilities was fairly balanced including 51.8% presenting to rural and 48.2% to urban facilities. About half of the patients with reported antibiotic use from BF (49.9%) and from RSA (52.1%) reported living in a village in contrast to 37.5% in CIV and 27.7% in DRC. Slightly more than half of enrolled patients with reported antibiotic use were children under the age of five (*n* = 4,077, 57.0%) and 45.9% were female. A greater proportion of adult patients or legal guardians of minors reported no education in CIV (*n* = 354, 53.7%) and BF (*n* = 1,388, 56.6%) compared to DRC (*n* = 315, 16.6%) and RSA (*n* = 42, 1.9%). Approximately half of the patients or legal guardians of minors who reported antibiotic use were unemployed, ranging from 21.5% (*n* = 495) in BF to 72.6% (*n* = 1,592) in RSA. Among those with data on hospitalization, 99.3% of the patients with reported antibiotic use were currently hospitalized at enrolment, although data on hospitalization was incomplete, particularly in CIV (45.4%, 299/659 missing entries) and DRC (19.9%, 378/1,903 missing entries) (see Table 1).

**Table 1:** Characteristics of study participants reporting antibiotic use ten days prior to study enrolment by country

	Country									
	Total (*N* = 7,258)		Côte d’Ivoire (*N* = 659)		Burkina Faso (*N* = 2,470)		DR Congo (*N* = 1,903)		Rep. South Africa (*N* = 2,226)	
	*n*	%	*n*	%	*n*	%	*n*	%	*n*	%
*Syndrome*										
AFDUC	2,198	30.3%	187	28.4%	879	35.6%	514	27.0%	618	27.8%
GI	1,642	22.6%	207	31.4%	355	14.4%	538	28.3%	542	24.3%
RTI	3,009	41.5%	261	39.6%	1,161	47.0%	665	34.9%	922	41.4%
GI/RTI	409	5.6%	4	0.6%	75	3.0%	186	9.8%	144	6.5%
*Covid-19 pandemic*										
Enrolled before	4,319	59.5%	508	77.1%	1,309	53.0%	1,181	62.1%	1,321	59.3%
Enrolled during	2,939	40.5%	151	22.9%	1,161	47.0%	722	37.9%	905	40.7%
*Health facility*										
Rural site	1,974	27.2%	86	13.1%	357	14.5%	379	19.9%	1,152	51.8%
Urban site	5,284	72.8%	573	86.9%	2,113	85.5%	1,524	80.1%	1,074	48.2%
Patient’s residence*	*N* = 7,212		*N* = 659		*N* = 2,451		*N* = 1,902		*N* = 2,200	
Village	3,142	43.6%	247	37.5%	1,222	49.9%	526	27.7%	1,147	52.1%
City/Town	4,070	56.4%	412	62.5%	1,229	50.1%	1,376	72.3%	1,053	47.9%
Age group*	*N* = 7,155		*N* = 659		*N* = 2,465		*N* = 1,899		*N* = 2,132	
<1 year	1,947	27.2%	101	15.3%	463	18.8%	721	38.0%	662	31.1%
1–4 years	2,130	29.8%	170	25.8%	634	25.7%	675	35.5%	651	30.5%
5–17 years	635	8.9%	61	9.3%	231	9.4%	206	10.8%	137	6.4%
18–44 years	1,269	17.7%	167	25.3%	535	21.7%	163	8.6%	404	18.9%
≥45 years	1,174	16.4%	160	24.3%	602	24.4%	134	7.1%	278	13.0%
Sex*	*N* = 7,243		*N* = 659		*N* = 2,463		*N* = 1,903		*N* = 2,218	
Male	3,916	54.1%	344	52.2%	1,458	59.2%	1,015	53.3%	1,099	49.5%
Female	3,327	45.9%	315	47.8%	1,005	40.8%	888	46.7%	1,119	50.5%
Level of education*	*N* = 7,215		*N* = 659		*N* = 2,452		*N* = 1,900		*N* = 2,204	
No level of education	2,099	29.1%	354	53.7%	1,388	56.6%	315	16.6%	42	1.9%
≤ 6 years	1,459	20.2%	114	17.3%	578	23.6%	576	30.3%	191	8.7%
7–10 years	1,712	23.7%	85	12.9%	334	13.6%	678	35.7%	615	27.9%
> 10 years	1,945	27.0%	106	16.1%	152	6.2%	331	17.4%	1,356	61.5%
Employment*	*N* = 7,042		*N* = 649		*N* = 2,304		*N* = 1,895		*N* = 2,194	
Unemployed	3,497	49.7%	271	41.8%	495	21.5%	1,139	60.1%	1,592	72.6%
Self-employed	2,140	30.4%	267	41.1%	1,327	57.6%	490	25.9%	56	2.6%
Part time employed	391	5.6%	16	2.5%	152	6.6%	113	6.0%	110	5.0%
Full time employed	1,014	14.4%	95	14.6%	330	14.3%	153	8.1%	436	19.9%
*Hospitalized at enrolment¥*	*N* = 6,447		*N* = 360		*N* = 2,344		*N* = 1,525		*N* = 2,218	
No	42	0.7%	3	0.8%	28	1.2%	10	0.7%	1	0.1%
Yes	6,405	99.3%	357	99.2%	2,316	98.8%	1,515	99.3%	2,217	99.9%

Legend: Enrolment period 1 February 2018 till 26 May 2022; AFDUC: acute febrile disease of unknown cause; GI: gastrointestinal infection; RTI: respiratory tract infection; DR Congo: Democratic Republic of the Congo; Rep. South Africa: Democratic Republic of South Africa; *Variables with missing data (< 5%); ¥Missing data exceeds 5%

Differences in characteristics of patients who did and did not report antibiotic use across all patients enrolled in the study period can be seen in Additional file 7.

Among the 7,258 patients who reported antibiotic use in the ten days prior to study enrolment, 3,009 (41.5%) were enrolled with RTI, 2,198 (30.3%) with AFDUC, and 1,642 (22.6%) with GI (Tables 1 and 2). Over 90% (*n* = 6,957) of patients who reported antibiotic use had taken the last dose of the reported antibiotic within 2 days prior to study enrolment. Across all syndromes, 45.7% (3,276/7,170) reported symptom onset in the 4–7 days prior to enrolment. Among those with the GI syndrome, 48.2% (757/1,569) reported onset of symptoms in the 0–3 days prior to enrolment.

Half of the patients (2,676/5,325, 50.5%) with available BMI/z-score data who reported antibiotic use were of normal weight, 32.8% (1,749/5,325) were underweight, and 16.9% (900/5,325) were overweight or obese. In particular, approximately half of patients with a GI or GI/RTI syndrome who reported antibiotic use were underweight (514/1,100, 46.7%; and 136/261, 52.1% respectively). Among those with treatment data, 20.0% (1,405/7,036) of patients who reported antibiotic use also reported use of antimalarials and 51.5% (3,601/6,992) reported use of other medication such as analgesics, dietary supplements, antiparasitic drugs (other than antimalarials) and corticosteroids, with only minor differences between the syndrome enrolment (Table 2).

**Table 2:** Characteristics of study participants reporting antibiotic use in the ten days prior to enrolment by syndrome

	Syndrome enrolment										
	Total^1^		AFDUC		GI		RTI		GI/RTI		
	*N* = 7,258		*N* = 2,198		*N* = 1,642		*N* = 3,009		*N* = 409		
	*n*	%	*n*	%	*n*	%	*n*	%	*n*	%	
*Last dose of reported antibiotic°*											
Same day	3,860	53.2%	1,188	54.0%	880	53.6%	1,619	53.8%	173	42.3%	
1–2 days ago	3,097	42.7%	916	41.7%	697	42.4%	1,272	42.3%	212	51.8%	
3–10 days ago	301	4.1%	94	4.3%	65	4.0%	118	3.9%	24	5.9%	
Symptom onset*	*N* = 7,170		*N* = 2,191		*N* = 1,569		*N* = 3,007		*N* = 403		
0–3 days ago	2,763	38.5%	881	40.2%	757	48.2%	996	33.1%	129	32.0%	
4–7 days ago	3,276	45.7%	942	43.0%	681	43.4%	1,421	47.3%	232	57.6%	
8–10 days ago	1,131	15.8%	368	16.8%	131	8.3%	590	19.6%	42	10.4%	
Sex and age*	*N* = 7,140		*N* = 2,142		*N* = 1,624		*N* = 2,970		*N* = 404		
Male, < 5 years	2,286	32.0%	524	24.5%	673	41.4%	882	29.7%	207	51.2%	
Female, < 5 years	1,784	25.0%	407	19.0%	495	30.5%	733	24.7%	149	36.9%	
Male, ≥ 5 years	1,578	22.1%	642	30.0%	192	11.8%	728	24.5%	16	4.0%	
Female, ≥ 5 years	1,492	20.9%	569	26.6%	264	16.3%	627	21.1%	32	7.9%	
*Weight category¥*	*N* = 5,325		*N* = 1,600		*N* = 1,100		*N* = 2,364		*N* = 261		
Underweight	1,749	32.8%	401	25.1%	514	46.7%	698	29.5%	136	52.1%	
Normal	2,676	50.3%	919	57.4%	420	38.2%	1,240	52.5%	97	37.2%	
Overweight	569	10.7%	185	11.6%	99	9.0%	264	11.2%	21	8.0%	
Obese	331	6.2%	95	5.9%	67	6.1%	162	6.9%	7	2.7%	
Co-morbidities*	*N* = 7,181		*N* = 2,184		*N* = 1,627		*N* = 2,964		*N* = 406		
No	6,612	92.1%	2,017	92.4%	1,546	95.0%	2,676	90.3%	373	91.9%	
Yes	569	7.9%	167	7.6%	81	5.0%	288	9.7%	33	8.1%	
Antimalarials*	*N* = 7,036		*N* = 2,154		*N* = 1,600		*N* = 2,884		*N* = 398		
No	5,631	80.0%	1,684	78.2%	1,233	77.1%	2,423	84.0%	291	73.1%	
Yes	1,405	20.0%	470	21.8%	367	22.9%	461	16.0%	107	26.9%	
Other medication*	*N* = 6,992		*N* = 2,144		*N* = 1,581		*N* = 2,872		*N* = 395		
No	3,391	48.5%	1,029	48.0%	713	45.1%	1,506	52.4%	143	36.2%	
Yes	3,601	51.5%	1,115	52.0%	868	54.9%	1,366	47.6%	252	63.8%	

Legend 1: Enrolment from 1 February 2018 till 26 May 2022. AFDUC: acute febrile disease of unknown cause; GI: gastrointestinal infection; RTI: respiratory tract infection. ° only first reported antibiotic shown *Variables with missing data < 5%. ¥Missing data exceeds 5%. ^*1*^ *For a full row frequency table indicating characteristics of study participants who reported antibiotic use by syndrome see Additional file* 8

### Most commonly reported antibiotics

**Fig. 1:**
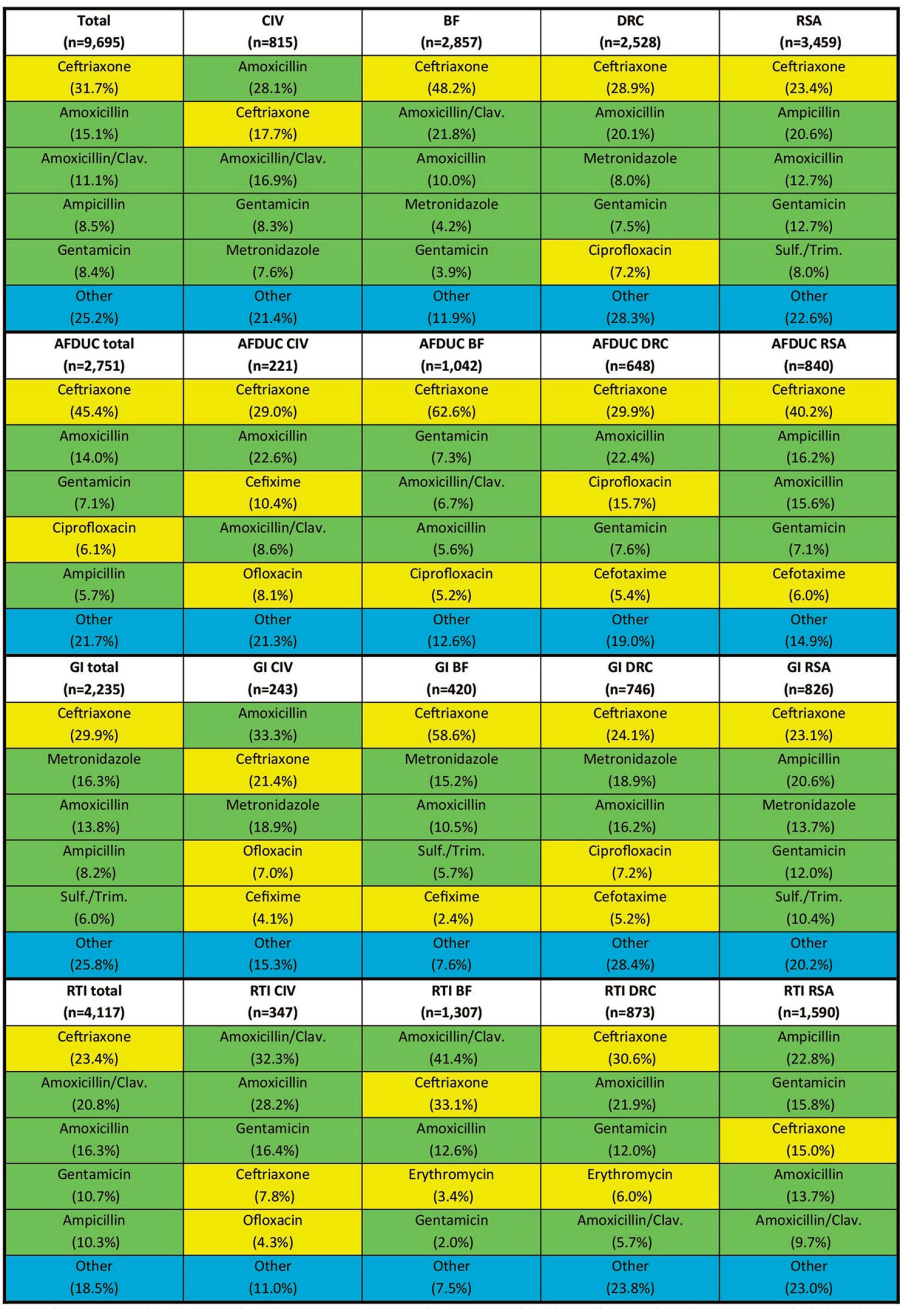
Top five most commonly reported antibiotics overall and by country, syndrome and WHO AWaRe classification. Legend: Access antibiotics are colored in green, Watch antibiotics are colored in yellow, and antibiotics not included in the top five reported antibiotics are grouped as “other” and colored in blue. Sulf./Trim. = Sulfamethoxazole/Trimethoprim; Amoxicillin/Clav. = Amoxicillin/clavulanic acid*. AFDUC: acute febrile disease of unknown cause; GI: gastrointestinal infection; RTI: respiratory tract infection; Combination of GI/RTI cases not shown separately (n = 592). CIV: Côte d’Ivoire, BF: Burkina Faso, DRC: The Democratic Republic of the Congo, RSA: The Republic of South Africa

Among 7,258 patients who reported antibiotic use, 9,695 antibiotics were reported in the ten days prior to study enrolment. The most common antibiotic reported was ceftriaxone (31.7%, *n* = 3,071), a WHO Watch antibiotic, followed by amoxicillin (15.1%, *n* = 1,466) and amoxicillin/clavulanic acid (11.1%, *n* = 1,075), both WHO Access antibiotics. Among patients with the AFDUC syndrome, ceftriaxone was the most commonly reported antibiotic, ranging from 29.0% in CIV (64/221) to 62.6% (652/1,042) in BF. Among patients with GI, ceftriaxone (ranging from 21.4% (52/243) in CIV to 58.6% (246/420) in BF), and metronidazole (ranging from 13.7% (113/826) in RSA to 18.9% in DRC and CIV (141/746 and 46/243 respectively) were the most commonly reported antibiotics. Among those with RTI, amoxicillin/clavulanic acid was the most commonly reported antibiotic in CIV (32.3%; 112/347) and BF (41.4%; 541/1,307). Apart from ceftriaxone, other Watch group antibiotics such as ciprofloxacin (in particular, 6.1% (169/2,751) of patients with AFDUC), cefotaxime, cefixime, ofloxacin and erythromycin were reported (Fig. 1). In total, only three (0.03%) carbapenems (imipenem once, meropenem twice) were reported. Of the 66 reported antibiotics belonging to the Not recommended group, 63 (95.5%) were reported from DRC. A majority of the 63 Not recommended group antibiotics reported in DRC came from the fixed-dose-combination metronidazole/norfloxacin (71.4% or 45/63) and primarily among patients with the GI and GI/RTI syndrome (66.7% or 42/63).

**Fig. 2:**
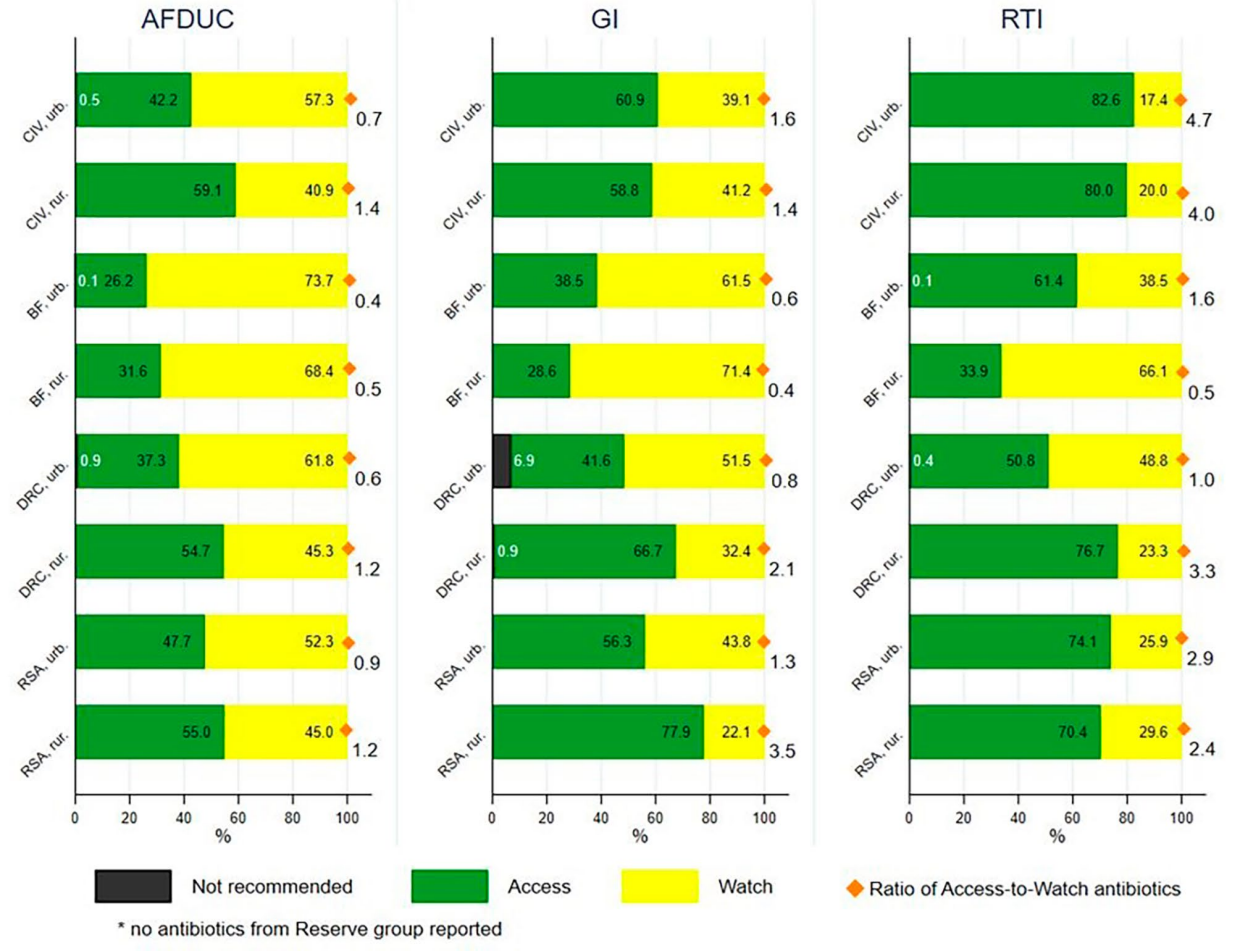
Proportional antibiotic use according to WHO AWaRe classification by syndrome, country and health facility location. *Legend: AFDUC: acute fever of unknown cause; GI: gastrointestinal infection; RTI: respiratory tract infection; CIV: Ivory Coast; BF: Burkina Faso; DRC: The Democratic Republic of the Congo; RSA: Republic of South Africa; urb.: urban health centre; rur.: rural health centre*

Among the top five antibiotics reported across countries, only Access and Watch antibiotics were found. Out of all 9,695 reported antibiotics, 54.7% (*n* = 5,299) were Access, 44.7% (*n* = 4,330) were Watch, 0.7% (*n* = 66) were Not recommended, and 0% (*n* = 0) were Reserve group antibiotics. The overall ratio of Access to Watch antibiotics was 1.2. This varied by country, urban and rural health facilities and syndrome, (Fig. 2) ranging from 0.4 among rural facilities in BF to 4.7 among urban facilities in CIV. Among those with the AFDUC syndrome, approximately half or less of reported antibiotics were Access antibiotics. Among those with the GI syndrome, a greater number of patient reported antibiotics were Access antibiotics, especially across the urban health facilities in CIV and rural health care facilities in DRC and RSA which reported greater than 60% of Access group antibiotics. Among those with the RTI syndrome, the greatest number of Access antibiotics were.

reported including all health care facilities in CIV and RSA as well as rural facilities in DRC reporting more than 70% Access antibiotics (Fig. 2).

Before the COVID-19 pandemic, 40.7% (2,352/5,783) of all reported antibiotics were from the Watch group. This increased to 50.6% (1,978/3,912) during the pandemic (see Additional File 9 for these results by country). The relative increase in the reported use of Watch antibiotics during the pandemic were most pronounced among those with the AFDUC syndrome, from 54.4% (809/1,487) reported antibiotics in AFDUC before the pandemic to 67.6% (855/1,264) during the pandemic, although results varied by country and location. The increase in the reported use of Watch antibiotics from before to during the pandemic were less pronounced among those with GI and RTI, although differences by country and location were also present, particularly in rural RSA where the reported use of Watch among patients with RTI went from 17.3% (84/485) before the pandemic to 42.3% (200/473) during the pandemic.

Due to a missing or an invalid reported date of the last dose, 611 reported antibiotics were excluded from the main analysis. Among these excluded antibiotics, metronidazole was reported in 47.6% (*n* = 291). A sensitivity analysis of the most commonly reported antibiotics by country and WHO AWaRe classification including these additional antibiotics (*n* = 10,306) is reported in the supplementary text (see Additional File 10).

### Routes of administration and number of reported antibiotics

Of 9,695 total antibiotics reported, 51.6% (*n* = 4,998) were classified as parenteral use only. Across patients in all countries and by syndrome, these antibiotics included most commonly ceftriaxone, gentamicin, cefotaxime and ampicillin. In BF, ceftriaxone accounted for 90.2% (1,378/1,527) of reported antibiotics classified as parenteral formulations. Of the parenteral classified antibiotics, the last dose was reported on the same day of study enrollment in 52.3% (*n* = 2,615) of cases, one day prior in 41.9% (*n* = 2,095) and 3–10 days prior in 5.8% (*n* = 288) of cases.

The number of different antibiotic substances reported varied considerably between countries and between urban or rural location of the health facilities. While urban DRC reported 37 different antibiotic substances, of which 12 were classified as parenteral, rural CIV, BF and DRC each reported 14 different antibiotic substances, of which two (rural CIV) and four (rural BF and rural DRC) were classified as parenteral formulations (see Additional File 11).

**Fig. 3:**
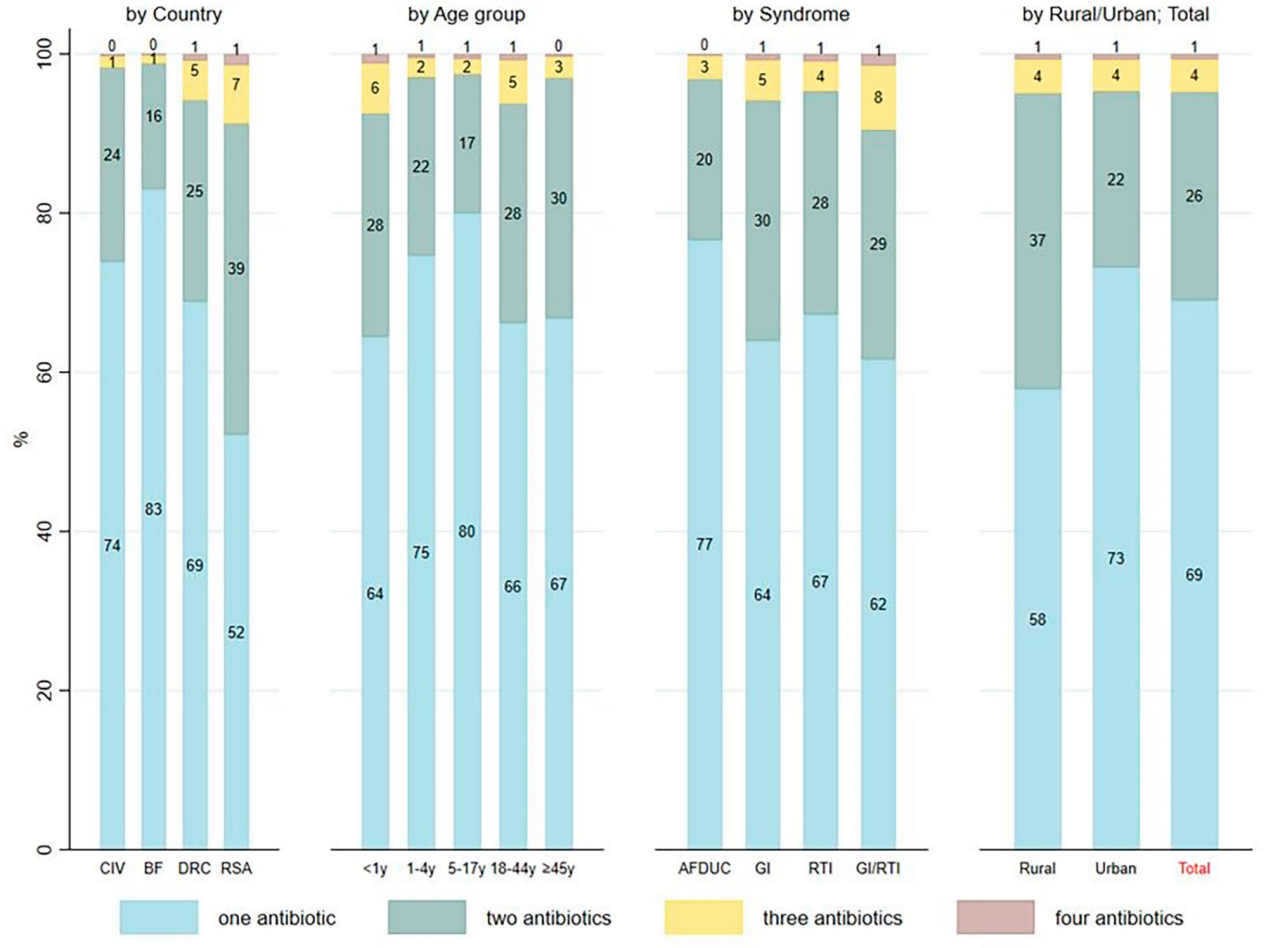
Number of antibiotics reported per patient in the ten days prior to study inclusion. *Legend: CIV: Côte d’Ivoire; BF: Burkina Faso; DRC: Democratic Republic of the Congo; RSA: Republic of South Africa; AFDUC: acute fever of unknown cause; GI: gastrointestinal infection; RTI: respiratory tract infection*

Among the 7,258 patients who received antibiotics, 69.0% (*n* = 5,010) reported the use of only one antibiotic, followed by 26.2% (*n* = 1,904) who received two antibiotics, and 4.7% (*n* = 344) had received three or four antibiotics. In BF, 83.0% (*n* = 2,051) reported only one antibiotic ten days prior to study enrolment, in contrast to patients in RSA where 52.2% (*n* = 1,161) reported only one antibiotic. Furthermore, more patients enrolled at urban health care facilities received only one antibiotic (73.2%, 5,284/3,867) before enrolment compared to those at rural health facilities (57.9%, 1,143/1,974) (Fig. 3).

The sensitivity analysis assessing the number of antibiotics reported among patients who reported antibiotic use on the same day as study enrolment only (as opposed to the full time period of ten days prior) showed similar trends.

## Discussion

We investigated the self-reported antibiotic use in the ten days prior to enrolment among patients in a large infectious disease surveillance study in sub-Saharan Africa. Use of WHO Watch antibiotics, particularly the third-generation cephalosporin ceftriaxone, was frequently reported. Despite the high burden of infectious diseases due to resistant bacteria in these countries suggested by other studies [1], no antibiotic from the Reserve group was reported. Importantly, Watch antibiotic use varied between the clinical syndromes of AFDUC, GI and RTI as well as by location of participating health facilities including country and urban or rural setting. In three ANDEMIA countries, relative use of Watch antibiotics increased during the COVID-19 pandemic.

A recent review and meta-analysis suggested that the selection of multidrug resistant bacteria is associated more with exposure to antibiotics from the Watch or Reserve groups compared to those from the Access group [30]. High rates of Watch group antibiotics in LMICs, including a large proportion of ceftriaxone, have been described in other antibiotic consumption or use studies [31–35], such as those using antibiotic sales data [5, 9] or point-prevalence surveys [33, 35]. Some of these studies have assessed antibiotic use according to the AWaRe criteria by fever (i.e. main symptom for triggering antibiotic use) [31, 32] and other clinical symptoms.

In this study, we focused on the defined infectious disease syndromes of AFDUC, GI and RTI used in the ANDEMIA surveillance study. These syndrome-specific self-reported antibiotic use findings provide an important contribution to the evidence base given the high burden of these infections in LMICs, where respiratory tract infections (including tuberculosis), enteric infections and other infectious diseases have been found to account for more than 30% of all total causes of deaths in children < 5 years [36, 37].

In our study, although patients with RTI most frequently reported overall antibiotic use (43.1%), the proportion of reported Watch antibiotic use was the lowest among this patient group, and reported antibiotics more frequently included those from the Access group. In contrast, the highest proportion of Watch antibiotic use was among AFDUC patients, including ceftriaxone as the most commonly reported antibiotic among AFUDC and GI patients.

Furthermore, there was a high proportion of reported use of ceftriaxone, a broad-spectrum beta-lactam in our study. It should be acknowledged that ceftriaxone is a relatively easy to administer and well-tolerated parenteral antibiotic. It has been off-patent since 2005 [38] and is generically available in all four ANDEMIA countries.

To guide the empiric use of antibiotics according to the AWaRe criteria, the recently published WHO AWaRe book and the earlier published WHO Essential Medicine List were developed to support treatment decision making [25, 39, 40]. Some of our syndrome-specific findings appeared to be consistent with the recommendations proposed by these two tools. Ceftriaxone is recommended as one of the first-choice antibiotics for severe community acquired pneumonia (CAP), severe enteric fever with risk for fluoroquinolone resistance, and acute bacterial meningitis. In our study, 6.1% of patients with reported antibiotic use presented with meningeal signs. Ceftriaxone is also recommended as a second-choice treatment for acute infectious diarrhea/gastroenteritis and for sepsis in neonates and children [25, 40], which should be considered in our study given that 57.0% of patients with reported antibiotic use were under the age of five.

Among RTI patients in our study, all five reported antibiotics were the first-, or second choice antibiotic for mild, moderate or severe CAP cases for adults or children according to the AWaRe book [40]. Nonetheless, overprescribing or self-medication of antibiotics in (upper) RTI and inappropriate use of ceftriaxone has been previously described also in LMIC settings [34, 41, 42].

Thus, assessments of antibiotic use by individual patient factors, severity of presentation, and if available, further diagnostics remains crucial to assess the appropriateness of antibiotic use in LMICs.

Another finding in the present study is a shift from Access to increased Watch antibiotic use during the COVID-19 pandemic for BF, DRC and RSA. A critical review comparing treatment guidelines for COVID-19 patients across ten different countries in Sub-Saharan Africa found that some guidelines recommended the use of Watch group antibiotics [43], although only one of the ten countries included was an ANDEMIA country (RSA). In addition, a decrease in antibiotic use per capita has been described during the COVID-19 pandemic [44] which is consistent with our findings, although other factors such as COVID-19 associated mitigation measures and lower attendance to hospitals especially in the beginning of the pandemic may also play a role [45]. Thus, confounding factors cannot be excluded and there may be a need for further studies on the influence of the COVID-19 pandemic and antibiotic consumption or use.

In our study, differences in overall reported antibiotic use were seen by country, ranging from 11.9% in CIV to more than half of enrolled patients in RSA and BF reporting antibiotic use. Furthermore, it was found that the overall reported Access antibiotic use in CIV and RSA was more than 60% compared to lower proportions in BF and in DRC. In addition to varying patient clinical presentation, and location of facilities, as well as slightly different enrolment in the countries, other programmatic aspects may also be considered when interpreting these findings. Differences in governance of AMR prevention and control strategies as well as structures for accountability, surveillance and financing across countries may play important roles [14, 46]. Although national action plans (NAPs) on AMR exist in all four ANDEMIA countries [47–49], implementation can pose significant challenges [46, 50, 51]. As demonstrated by this study, the WHO Access, Watch, and Reserve (AWaRe) classification system of antibiotics according to their spectrum of activity and potential to develop resistance offers an objective framework which can be used to guide evaluations of antibiotic use as well as inform the development and implementation of national policies on antimicrobial stewardship. At the health facility level, patients often presented to urban facilities even if their place of residence was in the village. Antibiotic use was more commonly reported from patients presenting at urban facilities, although multiple therapies were more often reported from patients presenting at rural facilities. Rural health facilities may be facing challenges with lack of staff, access to diagnostic tools and drug shortages [52], which may influence self-medication and switch of treatment, leading to several therapies. Also, across the ANDEMIA facilities different antibiotics reported ranged between 14 and 37 antibiotics (including fixed-dose combinations and WHO Not recommended antibiotics). Reasons for the use of Not recommended antibiotics may include limited access to individual antibiotic formulations and lack of enforced regulation [53].

In this study, 0.03% (*n* = 3) carbapenems (Watch group) and no antibiotics from the Reserve group were reported. This is particularly notable given that the recent global study modelling the burden of AMR estimated that the proportion of third-generation cephalosporin-resistant isolates for *Escherichia coli* ranged between 20 and 50% in BF, CIV, DRC and RSA, with even higher proportions of 60–80% resistant for *Klebsiella pneumoniae* and up to 70% carbapenem-resistant isolates *for Acinetobacter baumannii* (in RSA) [1].

As outlined above, antibiotic availability and accessibility must be considered when interpreting our results. A recent spatial modelling study comparing global antibiotic consumption data and surveys on antibiotic use found large variations of antibiotic usage also within LMICs, suggesting access and availability to be one explanation [54]. Stock-outs and drug shortages are well known in LMICs. A study which collected data on the availability and prices of drugs (including seven antibiotics) in government and church health facilities, private pharmacies as well as informal vendors in DRC and Cameroon found a wide variation in the availability of the antibiotics, ranging from 37% (antibiotic available at 12/34 facilities) to 94% (antibiotic available at 32/34 facilities) in DRC. This study also calculated the median price ratios and daily wages required for a full treatment course with each antibiotic. For DRC, the cost for a full treatment course ranged from 0.55 (doxycycline) to 10.05 (amoxicillin/clavulanic acid) of the equivalent median daily wage [55]. Another study from Ethiopia which calculated the cost of a full treatment course for different infectious diseases found that for treatment of community acquired pneumonia with ceftriaxone (using 1 g i.v. every 12 h for 7 days), the prices ranged between seven daily wages if the substance came from a public pharmacy (lowest median price, 0.5USD/single dose) to 56 daily wages if the substance was bought from a private pharmacy (highest median price, 1.2USD) [56].

In this context, namely concerning the rising antibiotic consumption globally with high proportional Watch group use, promoting universal health coverage (UHC), and addressing economic inequalities that may force patients to choose treatment options based on affordability and accessibility rather than medical necessity and appropriateness is crucial to combat AMR.

Several limitations of the present study should be considered. No data on the antibiotic start date, full duration and prescriber (if any) were available and data on hospitalization was incomplete especially for CIV and DRC so it was not possible to clearly distinguish outpatient versus inpatient treatment in the ten days prior to study enrolment. Furthermore, ANDEMIA patients were enrolled within 24 h of presentation to the health care facility, so antibiotics received after enrolment were not captured. This limited the ability to conduct a regression analysis to further assess the factors influencing antimicrobial use. The reported antibiotic data including name and date of last dose may have also been affected by recall bias or lack of documentation during the clinical interview and completion of questionnaire. Differences in these practices or limitations may have also occurred across participating study facilities although all surveillance personal received the same training materials. Namely, in RSA, it was reported that surveillance officers allowed enrolment and specimen collection for up to 48 h, which may have led to higher reported antibiotic use and/or multiple therapies. Approximations concerning the routes of administration (i.e. parenteral, oral, or both/other) must be interpreted with caution, as the actual data were not reported and these results were coded according to the provided antibiotic names, available guidelines and expert clinical opinion. Also, antibiotic dosages were not available, which limits comparison with large antibiotic consumption studies based on sales data such as Klein et al. [5, 9]. It was not possible to assess reported antibiotic use in special patient populations such as those with human immunodeficiency virus (HIV) or tuberculosis (Tb) as patient numbers across health care facility locations were too small. Finally, severity of illness and patient follow-up were not assessed in this study as the complete data for these variables were not available.

## Conclusion

Relatively high levels of Watch group antibiotic use, particularly in acute febrile disease of unknown cause and for gastrointestinal infections, pose a challenge to antibiotic use interventions to address the burden of AMR. A nuanced perspective on the clinical presentation of the patient, the country-context, accessibility, and affordability of care and treatment needs to be considered when planning and implementing strategies to reduce inappropriate Watch group antibiotic use.

### Electronic supplementary material

Below is the link to the electronic supplementary material.

Additional file 1. Table of relevant country data and study sites (.pdf).

Additional file 2. Figure on ANDEMIA case-definitions (.pdf).

Additional file 3. Question on antibiotics use, ANDEMIA case investigation form (.pdf).

Additional file 4. Table of the coding frameworks for the antibiotic formulation and WHO AWaRe criteria (.pdf).

Additional file 5. Table: Body Mass Index calculation (pdf).

Additional file 6. Table of characteristics of ANDEMIA patients enrolled from 1 February 2018 till 26 May 2022 by country (.pdf).

Additional file 7. Table of reported antibiotic use in the ten days prior to study enrolment in the study population (.docx).

Additional file 8. Table of syndrome enrolment of patients that reported antibiotic use in the ten days prior to study enrolment with row frequencies (.docx).

Additional file 9. Figure on proportional antibiotic use according to WHO AWaRe classification by country, before and during COVID-19 pandemic (.docx/.tif).

Additional file 10. Figure on total reported antibiotics regardless of date of last dose among ANDEMIA total as well as by country (.pdf).

Additional file 11. Table on the number of different antibiotic substances reported in the ANDEMIA study by country and by location (.pdf).

## Data Availability

All data generated or analysed during this study are included in this published article and its supplementary information files. Datasets used during the current study are available from the corresponding author on reasonable request.

## Abbreviations

AFDUC: Acute febrile disease of unknown cause
AMR: Antimicrobial resistance
ANDEMIA: African Network for Improved Diagnostics, Epidemiology and Management of Common Infectious Agents
AWaRe: Access, Watch, Reserve
BF: Burkina Faso
BMI: Body Mass Index
CAP: Community acquired pneumonia
CI: Confidence Interval
CIV: Côte d’Ivoire
DRC: Democratic Republic of the Congo
GCS: Glasgow Coma Scale
GI: Gastrointestinal infection
HIV: Human immunodeficiency virus
IQR: Interquartile range
LMICs: Low-and middle-income countries
PPS: Point-prevalence survey
RSA: Republic of South Africa
RTI: Acute respiratory infection
Tb: Tuberculosis
USD: United States Dollar
WaSH: Water, sanitation and hygiene
WHO: World Health Organization
