# Supplementary material for: Reported antibiotic use among patients in the multicenter ANDEMIA infectious diseases surveillance study in sub-saharan Africa
Source: Antimicrob Resist Infect Control. 2024 Jan 25;13:9. doi: 10.1186/s13756-024-01365-w (PMC10809765; doi:10.1186/s13756-024-01365-w)
Supplement: Supplementary file 1 — Additional file 1. Table of relevant country data and study sites (.pdf). [file 13756_2024_1365_MOESM1_ESM.pdf]

Additional file 1

Table: Relevant country data and study sites

Country	Côte d'Ivoire	Burkina Faso	Democratic Republic of the Congo	Republic of South Africa
Study sites	1) Bouaké<br>2) Brobo Hospital<br>3) Guiglo<br>4) Tai	1) Souro Sanou, Bobo-Dioulasso<br>2) Dano	1) Kingasani<br>2) Kipako; Kimuisi; Ngeba<br>3) Kisantu Saint Luc General Hospital; Cerphytoco<br>4) Lisungi	1) Kalafong<br>2) Mapulaneng<br>3) Matikwana
Type of health facility	1) Urban university teaching hospital<br>2) Rural health centre with maternity ward<br>3) Regional hospital<br>4) Rural health centre	1) Urban university teaching hospital<br>2) Rural health care centre	1) Urban hospital<br>2) Rural health centres<br>3) Regional general hospital and health care centre close by<br>4) Rural health care centre	1) Urban hospital<br>2) Regional hospital<br>3) Medium district hospital
Country population, 2020 (1)	26.38 Mio<br>51.7.6% in urban areas	20.90 Mio<br>30.6% in urban areas	89.56 Mio<br>45.6% in urban areas	59.31 Mio<br>67.4% in urban areas
Median age of the country's population (estimate) 2020 (2)	17.5	16.5	15.6	26.9
Country life expectancy at birth in years, 2020 (1)	58.1	62.0	61.0	64.4
Country Rank Human Development Index (1-191), 2020 (3)	159	185	180	102
Country GDP per capita, 2020 (1)	2,325.7	857.9	544.0	5,655.9
Socio-demographic Index (SDI) 2019 (4)	0.408<br>(Low SDI)	0.257<br>(Low SDI)	0.382<br>(Low SDI)	0.678<br>(Middle SDI)

Legend: GDP: Gross domestic product; SDI Reference quintiles: Low SDI: 0-0.0454743; Low-middle SDI: 0.454743- 0.607679; Middle SDI: 0.607679-0.689504; High-middle SDI: 0.689504-0.805129; High SDI: 0.805129-1; all from (4)

Bibliography

1. World Bank national accounts data, OECD National Accounts data files [Internet]. 2022. Available from: https://databank.worldbank.org/.

2. World Population Prospects, Online Edition. [Internet]. United Nations. 2022. Available from: https://population.un.org/wpp/Download/Standard/MostUsed/.

3. United Nations Development Programme. Human Development Report 2021-22. 2022. http://report.hdr.undp.org

4. Global Burden of Disease Collaborative Network. Global Burden of Disease Study 2019 Socio-Demographic Index 1950–2019. Seattle: Institute for Health Metrics and Evaluation: 2020. https://ghdx.healthdata.org/record/ihme-data/gbd-2019-socio-demographic-index-sdi-1950-2019
