# Supplementary material for: Reported antibiotic use among patients in the multicenter ANDEMIA infectious diseases surveillance study in sub-saharan Africa
Source: Antimicrob Resist Infect Control. 2024 Jan 25;13:9. doi: 10.1186/s13756-024-01365-w (PMC10809765; doi:10.1186/s13756-024-01365-w)
Supplement: Supplementary file 2 — Additional file 2. Figure on ANDEMIA case-definitions (.pdf). [file 13756_2024_1365_MOESM2_ESM.pdf]

Additional file 2

Figure: ANDEMIA case definitions (1)

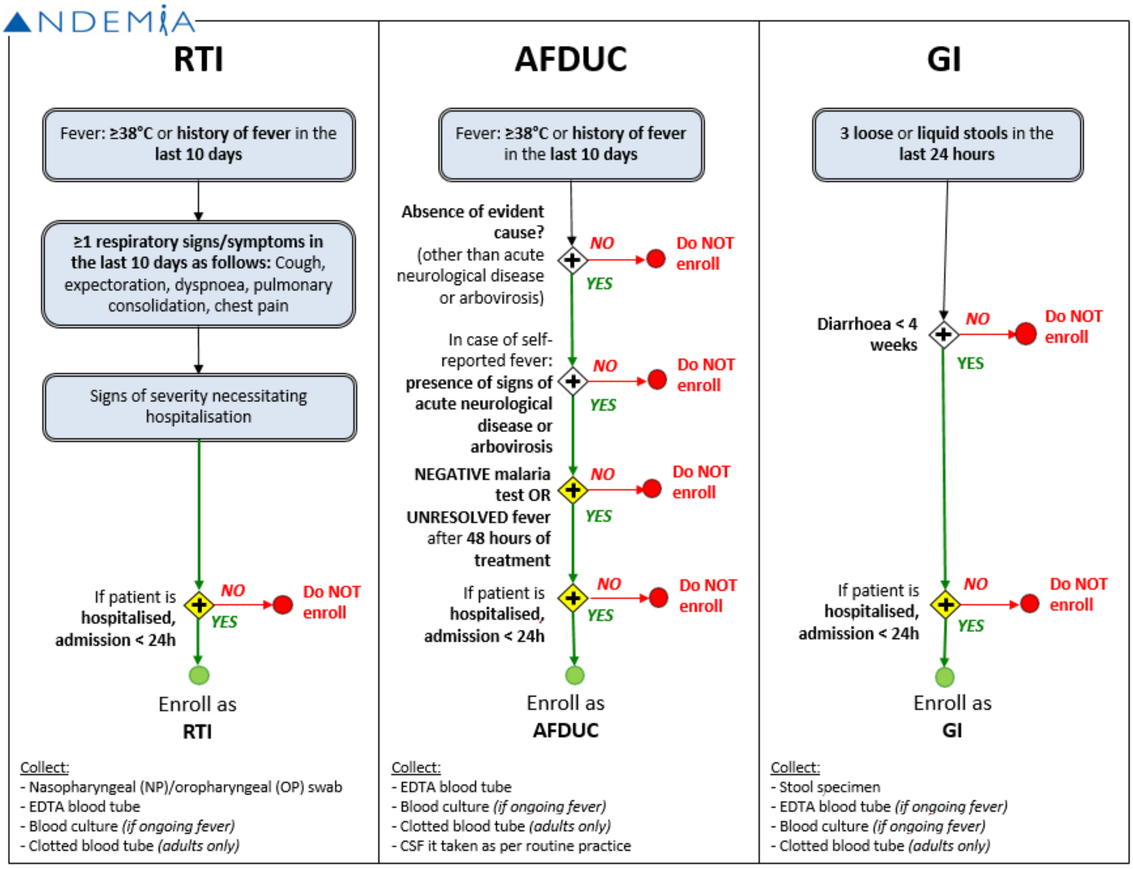

Legend: RTI: respiratory tract infection; AFDUC: acute febrile disease of unknown cause; GI: gastrointenstinal infection; EDTA: Ethylenediaminetetraacetic acid; CSF: cerebrospinal fluid

Bibliography

1. Schubert G, Achi V, Ahuka S, Belarbi E, Bourhaima O, Eckmanns T, et al. The African Network for Improved Diagnostics, Epidemiology and Management of common infectious Agents. BMC infectious diseases. 2021;21(1):539.
