# Supplementary material for: Reported antibiotic use among patients in the multicenter ANDEMIA infectious diseases surveillance study in sub-saharan Africa
Source: Antimicrob Resist Infect Control. 2024 Jan 25;13:9. doi: 10.1186/s13756-024-01365-w (PMC10809765; doi:10.1186/s13756-024-01365-w)
Supplement: Supplementary file 3 — Additional file 3. Question on antibiotics use, ANDEMIA case investigation form (.pdf). [file 13756_2024_1365_MOESM3_ESM.pdf]

# Additional file 3

Question on antibiotics use, ANDEMIA case investigation form

**Current treatment (prior to enrolment)**

*Please indicate any **treatment** taken in the **last 10 days** (other than malaria treatment, ART or TB treatment listed before). <u>Do **NOT** list medications prescribed at current consultation/admission</u> unless they have been taken before the collection of ANDEMIA samples.*

27. Is/has the patient taken any **antibiotics**? Yes ☐ No ☐

If **YES**, please specify below: (if **NO**, go to 28):

______________________________ date **last dose** taken: ☐☐/☐☐/20☐☐ (DD/MM/20YY)

______________________________ date **last dose** taken: ☐☐/☐☐/20☐☐ (DD/MM/20YY)

______________________________ date **last dose** taken: ☐☐/☐☐/20☐☐ (DD/MM/20YY)

______________________________ date **last dose** taken: ☐☐/☐☐/20☐☐ (DD/MM/20YY)

AMX=Amoxicilline; AMP=Ampicilline; AMC=Augmentin (Amoxicilline+Ac clavu.); CTX= Cefotaxime ; CFM=Cefixime; CIP=Ciprofloxacine; CLI=Clindamycine; CRO=Ceftriaxone; DOX=Doxycycline; ERY=Erythromycine; GEN=Gentamycine ; OFX=Ofloxacine ; PEN=Pénicilline; SXT=Cotrimoxazole; VAN=Vancomycine; Other (please specify in full above)
