# Supplementary material for: Reported antibiotic use among patients in the multicenter ANDEMIA infectious diseases surveillance study in sub-saharan Africa
Source: Antimicrob Resist Infect Control. 2024 Jan 25;13:9. doi: 10.1186/s13756-024-01365-w (PMC10809765; doi:10.1186/s13756-024-01365-w)
Supplement: Supplementary file 4 — Additional file 4. Table of the coding frameworks for the antibiotic formulation and WHO AWaRe criteria (.pdf). [file 13756_2024_1365_MOESM4_ESM.pdf]

Additional file 4

Table: The coding frameworks for the antibiotic formulation and WHO AWaRe criteria(1)

Antibiotic	WHO AWaRe	Formulation coded as:
Amikacin	Access	parenteral
Amoxicillin	Access	parenteral/oral/other*
Amoxicillin/clavulanic acid	Access	parenteral/oral/other*
Ampicillin	Access	parenteral
Azithromycin	Watch	oral
Cefadroxil	Access	oral
Cefazolin	Access	parenteral
Cefepime	Watch	parenteral
Cefepime/Tazobactam	Not recommended	parenteral
Cefixime	Watch	oral
Cefotaxime	Watch	parenteral
Cefpodoxime	Watch	oral
Ceftriaxone	Watch	parenteral
Ceftriaxone/Sulbactam	Not recommended	parenteral
Cefuroxime	Watch	parenteral/oral/other*
Cephalexin	Access	oral
Chloramphenicol	Access	parenteral/oral/other*
Ciprofloxacin	Watch	parenteral/oral/other*
Clarithromycin	Watch	parenteral/oral/other*
Clindamycin	Access	parenteral/oral/other*
Cloxacillin	Access	parenteral/oral/other*
Doxycycline	Access	parenteral/oral/other*
Erythromycin	Watch	parenteral/oral/other*
Flucloxacillin	Access	parenteral/oral/other*
Gentamicin	Access	parenteral
Levofloxacin	Watch	parenteral/oral/other*
Lincomycin	Watch	parenteral
Meropenem	Watch	parenteral
Metronidazole	Access	parenteral/oral/other*
Metronidazole/Norfloxacin	Not recommended	oral
Norfloxacin	Watch	oral
Ofloxacin	Watch	oral
Ofloxacin/Ornidazol	Not recommended	oral
Oxacillin	Access	parenteral/oral/other*
Penicillin	Access	parenteral/oral/other*
Piperacillin	Watch	parenteral
Rifampicin	Watch	oral
Sulfamethoxazole	Access	oral
Sulfamethoxazole/trimethoprim	Access	parenteral/oral/other
Tetracycline	Access	oral
Vancomycin	Watch	parenteral
Neomycin	Watch	parenteral/oral/other*
Piperacillin/Tazobactam	Watch	parenteral
Rifaximin	Watch	oral
Ticarcillin	Watch	parenteral
Tazobactam	Watch	parenteral
Imipenem	Watch	parenteral

Legend: Access antibiotics are colored in green; Watch antibiotics are colored in yellow; Not recommended antibiotics are colored in brown; No Reserve antibiotics are reported in the study; Antibiotic formulations coded as parenteral are colored in beige; Antibiotic formulations coded as oral are colored in light-blue; Parental antibiotic formulations coded as parenteral/oral/other are colored in light-green and refer to both (parenteral and oral) or other possible routes of administration.*

Bibliography

1. World Health Organization. WHO Access, Watch, Reserve (AWaRe) classification of antibiotics for evaluation and monitoring of use. Geneva: World Health Organization: 2021. https://www.who.int/publications/i/item/2021-aware-classification
