# Supplementary material for: Reported antibiotic use among patients in the multicenter ANDEMIA infectious diseases surveillance study in sub-saharan Africa
Source: Antimicrob Resist Infect Control. 2024 Jan 25;13:9. doi: 10.1186/s13756-024-01365-w (PMC10809765; doi:10.1186/s13756-024-01365-w)
Supplement: Supplementary file 5 — Additional file 5. Table: Body Mass Index calculation (pdf). [file 13756_2024_1365_MOESM5_ESM.pdf]

Additional file 5

Table: Weight category calculation

Only patients with valid height and weight data were analysed (1). The WHO anthro tool for BMI z score for 0-5 year old (2) and the Stata anthroplus tool for BMI z score calculation for 5-19 years (3) was used to calculate BMI z scores for the age groups 0-5 years and 5-19 years. Age specific categories were than reclassified to “underweight “normal” “overweight” and “obesity” to be comparable across the different age groups as shown in the following table.

Weight category	BMI/z-score 0-5years	BMI/z-score 5-19 years	BMI adult (KG/m^2)
Underweight	-4SD/- 2.01SD	Severe thinness (-4SD/-3SD)	Underweight (<18,5)
	-2SD/ -1.01SD	Thinness (<-2.99SD/-2.01)	
Normal	-1SD/0/+1SD	Normal (-2SD/0/+1SD)	Normal (18,5 / <25)
Overweight	1.01SD/2SD	Overweight (>1.01SD/2SD)	Overweight (25/ < 30)
Obesity	2.01SD/4SD	Obesity (2.01SD/4SD)	Obesity 1 (30/ <35)
			Obesity 2 (35/ <40)
			Obesity 3 (40+)

Legend: BMI: Body Mass Index; KG: Kilogram; SD: standard deviation.

Bibliography

1. World Health Organization. A healthy lifestyle - WHO recommendations. Europe 2010 6 May 2010. Contract No.: 10.08.2022. https://www.who.int/europe/news-room/fact-sheets/item/a-healthy-lifestyle---who-recommendations

2. World Health Organization. Child growth standards 2023 [The WHO Anthro Survey Analyser, anthro tool for BMI z score for 0-5 year old]. Available from: https://www.who.int/tools/child-growth-standards/software.

3. World Health Organization. Growth reference data for 5-19 years 2023 [Stata anthroplus tool for BMI z score calculation for 5-19 years]. Available from: https://www.who.int/tools/growth-reference-data-for-5to19-years/application-tools.
