# Supplementary material for: Reported antibiotic use among patients in the multicenter ANDEMIA infectious diseases surveillance study in sub-saharan Africa
Source: Antimicrob Resist Infect Control. 2024 Jan 25;13:9. doi: 10.1186/s13756-024-01365-w (PMC10809765; doi:10.1186/s13756-024-01365-w)
Supplement: Supplementary file 6 — Additional file 6. Table of characteristics of ANDEMIA patients enrolled from 1 February 2018 till 26 May 2022 by country (.pdf). [file 13756_2024_1365_MOESM6_ESM.pdf]

Additional file 6

Table: Characteristics of ANDEMIA case-patients enrolled from 1 February 2018 till 26 May 2022 by country

	Country									
	Total (N=19,700)		Côte d'Ivoire (N=5,529)		Burkina Faso (N=4,802)		DR Congo (N=5,937)		Rep. South Africa (N=3,432)	
	n	%	n	%	n	%	n	%	n	%
Syndrome										
AFDUC	7,203	36.6%	2,247	40.6%	1,784	37.2%	2,110	35.5%	1,062	30.9%
GI	5,085	25.8%	1,392	25.2%	1,334	27.8%	1,382	23.3%	977	28.5%
RTI	6,676	33.9%	1,820	32.9%	1,576	32.8%	2,075	35.0%	1,205	35.1%
GI and RTI	736	3.7%	70	1.3%	108	2.2%	370	6.2%	188	5.5%
COVID-19 pandemic										
Enrolled before	10,604	53.8%	3,392	61.3%	2,047	42.6%	3,238	54.5%	1,927	56.1%
Enrolled during	9,096	46.2%	2,137	38.7%	2,755	57.4%	2,699	45.5%	1,505	43.9%
Health facility										
Rural site	7,173	36.4%	2,010	36.4%	1,999	41.6%	1,657	27.9%	1,507	43.9%
Urban site	12,527	63.6%	3,519	63.6%	2,803	58.4%	4,280	72.1%	1,925	56.1%
*Patient's residence **	*N=19,617*		*N=5,529*		*N=4,770*		*N=5,936*		*N=3,382*	
Village	8,624	44.0%	2,083	37.7%	3,104	65.1%	1,954	32.9%	1,483	43.9%
City/Town	10,993	56.0%	3,446	62.3%	1,666	34.9%	3,982	67.1%	1,899	56.1%
*Age group**	*N=19,544*		*N=5,517*		*N=4,783*		*N=5,925*		*N=3,319*	
<1 year	4,297	22.0%	675	12.2%	1,064	22.2%	1,477	24.9%	1,081	32.6%
1-4 years	6,128	31.4%	1,492	27.0%	1,573	32.9%	1,952	32.9%	1,111	33.5%
5-17 years	2,518	12.9%	825	15.0%	395	8.3%	1,075	18.1%	223	6.7%
18-44 years	4,067	20.8%	1,687	30.6%	956	20.0%	868	14.6%	556	16.8%
≥45 years	2,534	13.0%	838	15.2%	795	16.6%	553	9.3%	348	10.5%
*Sex**	*N=19,672*		*N=5,528*		*N=4,790*		*N=5,936*		*N=3,418*	
Male	10,116	51.4%	2,643	47.8%	2,706	56.5%	2,985	50.3%	1,782	52.1%
Female	9,556	48.6%	2,885	52.2%	2,084	43.5%	2,951	49.7%	1,636	47.9%
*Level of education **	*N=19,625*		*N=5,529*		*N=4,770*		*N=5,933*		*N=3,393*	
None	7,092	36.1%	2,987	54.0%	3,166	66.4%	876	14.8%	63	1.9%
≤6 years	3,852	19.6%	998	18.1%	871	18.3%	1,737	29.3%	246	7.3%
7-10 years	4,136	21.1%	710	12.8%	489	10.3%	2,061	34.7%	876	25.8%
>10 years	4,545	23.2%	834	15.1%	244	5.1%	1,259	21.2%	2,208	65.1%
*Employment**	*N=19,331*		*N=5,465*		*N=4,584*		*N=5,911*		*N=3,371*	
Unemployed	8,819	45.6%	2,381	43.6%	820	17.9%	3,250	55.0%	2,368	70.2%
Self-employed	6,912	35.8%	2,163	39.6%	3,051	66.6%	1,609	27.2%	89	2.6%
Part time employed	966	5.0%	139	2.5%	201	4.4%	435	7.4%	191	5.7%
Full time employed	2,634	13.6%	782	14.3%	512	11.2%	617	10.4%	723	21.4%
Hospitalized at enrolment¥	*N=11,168*		*N=1,334*		*N=3,394*		*N=3,128*		*N=3,312*	
No	159	1.4%	8	0.6%	124	3.7%	25	0.8%	2	0.1%
Yes	11,009	98.6%	1,326	99.4%	3,270	96.4%	3,103	99.2%	3,310	99.9%

*Legend: AFDUC: acute febrile disease of unknown cause; GI: gastrointestinal infection; RTI: respiratory tract infection; DR Congo: Democratic Republic of the Congo; Rep. South Africa: Democratic Republic of South Africa; *Variables with missing data <5%. ¥Missing data exceeds 5%.*
