# Supplementary material for: Reported antibiotic use among patients in the multicenter ANDEMIA infectious diseases surveillance study in sub-saharan Africa
Source: Antimicrob Resist Infect Control. 2024 Jan 25;13:9. doi: 10.1186/s13756-024-01365-w (PMC10809765; doi:10.1186/s13756-024-01365-w)
Supplement: Supplementary file 7 — Additional file 7. Table of reported antibiotic use in the ten days prior to study enrolment in the study population (.docx). [file 13756_2024_1365_MOESM7_ESM.docx]

# Additional file 7

Table: Reported antibiotic use in the ten days prior to study enrolment in the study population

|  | Total | Antibiotic use reported | |  |
| --- | --- | --- | --- | --- |
| Characteristics | N | n | % | p-value¥ |
| *Overall* | 19,700 | 7,258 | 36.8% |  |
| *Country* |  |  |  | <0.001 |
| CIV | 5,529 | 659 | 11.9% |  |
| BF | 4,802 | 2,470 | 51.4% |  |
| DRC | 5,937 | 1,903 | 32.1% |  |
| RSA | 3,432 | 2,226 | 64.9% |  |
| *Syndrome* |  |  |  | <0.001 |
| AFDUC | 7,203 | 2,198 | 30.5% |  |
| GI | 5,085 | 1,642 | 32.3% |  |
| RTI | 6,676 | 3,009 | 45.1% |  |
| GI/RTI | 736 | 409 | 55.6% |  |
| *Covid-19 pandemic* |  |  |  | <0.001 |
| Enrolled before | 10,604 | 4,319 | 40.7% |  |
| Enrolled during | 9,096 | 2,939 | 32.3% |  |
| *Health facility* |  |  |  | <0.001 |
| Rural site | 7,173 | 1,974 | 27.5% |  |
| Urban site | 12,527 | 5,284 | 42.2% |  |
| *Patient’s residence* (N=19,617)* |  |  |  | 0.395 |
| Village | 8,624 | 3,142 | 36.4% |  |
| City/Town | 10,993 | 4,070 | 37.0% |  |
| *Age group* (N=19,544)* |  |  |  | <0.001 |
| <1 year | 4,297 | 1,947 | 45.3% |  |
| 1-4 years | 6,128 | 2,130 | 34.8% |  |
| 5-17 years | 2,518 | 635 | 25.2% |  |
| 18-44 years | 4,067 | 1,269 | 31.2% |  |
| ≥45 years | 2,534 | 1,174 | 46.3% |  |
| *Sex* (N=19,672)* |  |  |  | <0.001 |
| Male | 10,116 | 3,916 | 38.7% |  |
| Female | 9,556 | 3,327 | 34.8% |  |
| *Level of education* (N=19,625)* |  |  |  | <0.001 |
| No level of education | 7,092 | 2,099 | 29.6% |  |
| ≤ 6 years | 3,852 | 1,459 | 37.9% |  |
| 7-10 years | 4,136 | 1,712 | 41.4% |  |
| > 10 years | 4,545 | 1,945 | 42.8% |  |
| *Employment* (N=19,331)* |  |  |  | <0.001 |
| Not working | 8,819 | 3,497 | 39.7% |  |
| Self-employed | 6,912 | 2,140 | 31.0% |  |
| Part time employed | 966 | 391 | 40.5% |  |
| Full time employed | 2,634 | 1,014 | 38.5% |  |
| *Weight categories* (N=13,051)** |  |  |  | <0.001 |
| Underweight | 3,920 | 1,749 | 44.6% |  |
| Normal | 6,594 | 2,676 | 40.6% |  |
| Overweight | 1,606 | 569 | 35.4% |  |
| Obese | 931 | 331 | 35.6% |  |
| *Comorbidities* (N=19,575)* |  |  |  | <0.001 |
| No | 18,378 | 6,612 | 36.0% |  |
| Yes | 1,197 | 569 | 47.5% |  |
| *Antimalarial use* (N=19,265)* |  |  |  | <0.001 |
| No | 16,626 | 5,631 | 33.9% |  |
| Yes | 2,639 | 1,405 | 53.2% |  |
| *Other medication use* (N=19,220)* |  |  |  | <0.001 |
| No | 12,155 | 3,391 | 27.9% |  |
| Yes | 7,065 | 3,601 | 51.0% |  |
| *Symptom onset* (N=19,573)* |  |  |  | <0.001 |
| 0-3 days earlier | 9,923 | 2,763 | 27.8% |  |
| 4-7 days earlier | 7,391 | 3,276 | 44.3% |  |
| 8-10 days earlier | 2,259 | 1,131 | 50.1% |  |

Legend: Enrolment from 1 February 2018 till 26 May 2022; AFDUC: acute febrile disease of unknown cause; GI: gastrointestinal infection; RTI: respiratory tract infection; *Variables with missing data <5%. **Missing data exceeds 5%. ¥ Pearson Chi-squared test used to determine p-value, although results should be interpreted with caution as the reference group of “no reported antibiotics” could include those who received antibiotics after the initial study enrollment period of 24-48 hours after presentation. IQR: Interquartile range
