# Supplementary material for: Reported antibiotic use among patients in the multicenter ANDEMIA infectious diseases surveillance study in sub-saharan Africa
Source: Antimicrob Resist Infect Control. 2024 Jan 25;13:9. doi: 10.1186/s13756-024-01365-w (PMC10809765; doi:10.1186/s13756-024-01365-w)
Supplement: Supplementary file 8 — Additional file 8. Table of syndrome enrolment of patients that reported antibiotic use in the ten days prior to study enrolment with row frequencies (.docx). [file 13756_2024_1365_MOESM8_ESM.docx]

# Additional file 8

Table: Syndrome enrolment of case-patients that reported antibiotic use in the ten days prior to study enrolment

|  | | **Syndrome enrolment** | | | | | | | | | | | | | |  |  |  |
| --- | --- | --- | --- | --- | --- | --- | --- | --- | --- | --- | --- | --- | --- | --- | --- | --- | --- | --- |
|  |  | | | | AFUDC | | | GI | | | RTI | | | GI/RTI | | | |  |
|  | | | Total | n | | % | n | | % | n | | % | n | | % | |  |  |
| *Health facility by country* | | |  |  | |  |  | |  |  | |  |  | |  | |  |  |
| CIV | | | 659 | 187 | | 28.4% | 207 | | 31.4% | 261 | | 39.6% | 4 | | 0.6% | |  |  |
| BF | | | 2,470 | 879 | | 35.6% | 355 | | 14.4% | 1,161 | | 47.0% | 75 | | 3.0% | |  |  |
| DRC | | | 1,903 | 514 | | 27.0% | 538 | | 28.3% | 665 | | 34.9% | 186 | | 9.8% | |  |  |
| RSA | | | 2,226 | 618 | | 27.8% | 542 | | 24.4% | 922 | | 41.4% | 144 | | 6.5% | |  |  |
| *Health facility by location* | | |  |  | |  |  | |  |  | |  |  | |  | |  |  |
| Rural site | | | 1,974 | 561 | | 28.4% | 541 | | 27.4% | 785 | | 39.8% | 87 | | 4.4% | |  |  |
| Urban site | | | 5,284 | 1,637 | | 31.0% | 1,101 | | 20.8% | 2,224 | | 42.1% | 322 | | 6.1% | |  |  |
| *Health facility by country/location* | | |  |  | |  |  | |  |  | |  |  | |  | |  |  |
| CIV, urban | | | 573 | 167 | | 29.1% | 164 | | 28.6% | 238 | | 41.5% | 4 | | 0.7% | |  |  |
| CIV, rural | | | 86 | 20 | | 23.3% | 43 | | 50.0% | 23 | | 26.7% | 0 | | 0.0% | |  |  |
| BF, urban | | | 2,113 | 716 | | 33.9% | 266 | | 12.6% | 1,061 | | 50.2% | 70 | | 3.3% | |  |  |
| BF, rural | | | 357 | 163 | | 45.7% | 89 | | 24.9% | 100 | | 28.0% | 5 | | 1.4% | |  |  |
| DRC, urban | | | 1,524 | 345 | | 22.6% | 444 | | 29.1% | 560 | | 36.7% | 175 | | 11.5% | |  |  |
| DRC, rural | | | 379 | 169 | | 44.6% | 94 | | 24.8% | 105 | | 27.7% | 11 | | 2.9% | |  |  |
| RSA, urban | | | 1,074 | 409 | | 38.1% | 227 | | 21.1% | 365 | | 34.0% | 73 | | 6.8% | |  |  |
| RSA, rural | | | 1,152 | 209 | | 18.1% | 315 | | 27.3% | 557 | | 48.4% | 71 | | 6.2% | |  |  |
| *COVID-19 pandemic* | | |  |  | |  |  | |  |  | |  |  | |  | |  |  |
| Enrolled before | | | 4,319 | 1,167 | | 27.0% | 956 | | 22.1% | 1,926 | | 44.6% | 270 | | 6.3% | |  |  |
| Enrolled during | | | 2,939 | 1,031 | | 35.1% | 686 | | 23.3% | 1,083 | | 36.9% | 139 | | 4.7% | |  |  |
| *Sex, by age*  *< or ≥ 5 years** | | |  |  | |  |  | |  |  | |  |  | |  | |  |  |
| Male, <5 years | | | 2,286 | 524 | | 22.9% | 673 | | 29.4% | 882 | | 38.6% | 207 | | 9.1% | |  |  |
| Female, <5 years | | | 1,784 | 407 | | 22.8% | 495 | | 27.7% | 733 | | 41.1% | 149 | | 8.4% | |  |  |
| Male, ≥5 years | | | 1,578 | 642 | | 40.7% | 192 | | 12.2% | 728 | | 46.1% | 16 | | 1.0% | |  |  |
| Female, ≥5 years | | | 1,492 | 569 | | 38.1% | 264 | | 17.7% | 627 | | 42.0% | 32 | | 2.1% | |  |  |
| *Weight categories*¥ | | |  |  | |  |  | |  |  | |  |  | |  | |  |  |
| Underweight | | | 1,749 | 401 | | 22.9% | 514 | | 29.4% | 698 | | 39.9% | 136 | | 7.8% | |  |  |
| Normal | | | 2,676 | 919 | | 34.3% | 420 | | 15.7% | 1,240 | | 46.3% | 97 | | 3.6% | |  |  |
| Overweight | | | 569 | 185 | | 32.5% | 99 | | 17.4% | 264 | | 46.4% | 21 | | 3.7% | |  |  |
| Obese | | | 331 | 95 | | 28.7% | 67 | | 20.2% | 162 | | 48.9% | 7 | | 2.1% | |  |  |
| *Co-mobidities* * | | |  |  | |  |  | |  |  | |  |  | |  | |  |  |
| No | | | 6,612 | 2,017 | | 30.5% | 1,546 | | 23.4% | 2,676 | | 40.5% | 373 | | 5.6% | |  |  |
| Yes | | | 569 | 167 | | 29.3% | 81 | | 14.2% | 288 | | 50.6% | 33 | | 5.8% | |  |  |
| *Antimalarials* * | | |  |  | |  |  | |  |  | |  |  | |  | |  |  |
| No | | | 5,631 | 1,684 | | 29.9% | 1,233 | | 21.9% | 2,423 | | 43.0% | 291 | | 5.2% | |  |  |
| Yes | | | 1,405 | 470 | | 33.5% | 367 | | 26.1% | 461 | | 32.8% | 107 | | 7.6% | |  |  |
| *Other medication** | | |  |  | |  |  | |  |  | |  |  | |  | |  |  |
| No | | | 3,391 | 1,029 | | 30.3% | 713 | | 21.0% | 1,506 | | 44.4% | 143 | | 4.2% | |  |  |
| Yes | | | 3,601 | 1,115 | | 31.0% | 868 | | 24.1% | 1,366 | | 37.9% | 252 | | 7.0% | |  |  |
| *Symptom onset** | | |  |  | |  |  | |  |  | |  |  | |  | |  |  |
| 0-3 days ago | | | 2,763 | 881 | | 31.9% | 757 | | 27.4% | 996 | | 36.0% | 129 | | 4.7% | |  |  |
| 4-7 days ago | | | 3,276 | 942 | | 28.8% | 681 | | 20.8% | 1,421 | | 43.4% | 232 | | 7.1% | |  |  |
| 8-10 days ago | | | 1,131 | 368 | | 32.5% | 131 | | 11.6% | 590 | | 52.2% | 42 | | 3.7% | |  |  |
| *Last dose rep. antibiotic°* | | |  |  | |  |  | |  |  | |  |  | |  | |  |  |
| same day | | | 3,860 | 1,188 | | 30.8% | 880 | | 22.8% | 1,619 | | 41.9% | 173 | | 4.5% | |  |  |
| 1-2 days ago | | | 3,097 | 916 | | 29.6% | 697 | | 22.5% | 1,272 | | 41.1% | 212 | | 6.8% | |  |  |
| 3-10 days ago | | | 301 | 94 | | 31.2% | 65 | | 21.6% | 118 | | 39.2% | 24 | | 8.0% | |  |  |

Legend: enrolment from 1 February 2018 till 26 May 2022. CIV: Côte d'Ivoire; BF: Burkina Faso; DRC: Democratic Republic of the Congo; RSA: Republic of South Africa; AFDUC: acute febrile disease of unknown cause; GI: gastrointestinal infection; RTI: respiratory tract infection. *Variables with missing data <5%. ¥Missing data exceeds 5%. °only first reported antibiotic shown ^1^Frequencies relate to row variables
