# Supplementary material for: Reported antibiotic use among patients in the multicenter ANDEMIA infectious diseases surveillance study in sub-saharan Africa
Source: Antimicrob Resist Infect Control. 2024 Jan 25;13:9. doi: 10.1186/s13756-024-01365-w (PMC10809765; doi:10.1186/s13756-024-01365-w)
Supplement: Supplementary file 9 — Additional file 9. Figure on proportional antibiotic use according to WHO AWaRe classification by country, before and during COVID-19 pandemic (.docx/.tif). [file 13756_2024_1365_MOESM9_ESM.docx]

# Additional file 9

Figure: Proportional antibiotic use according to WHO AWaRe classification by country, before and during COVID-19 pandemic


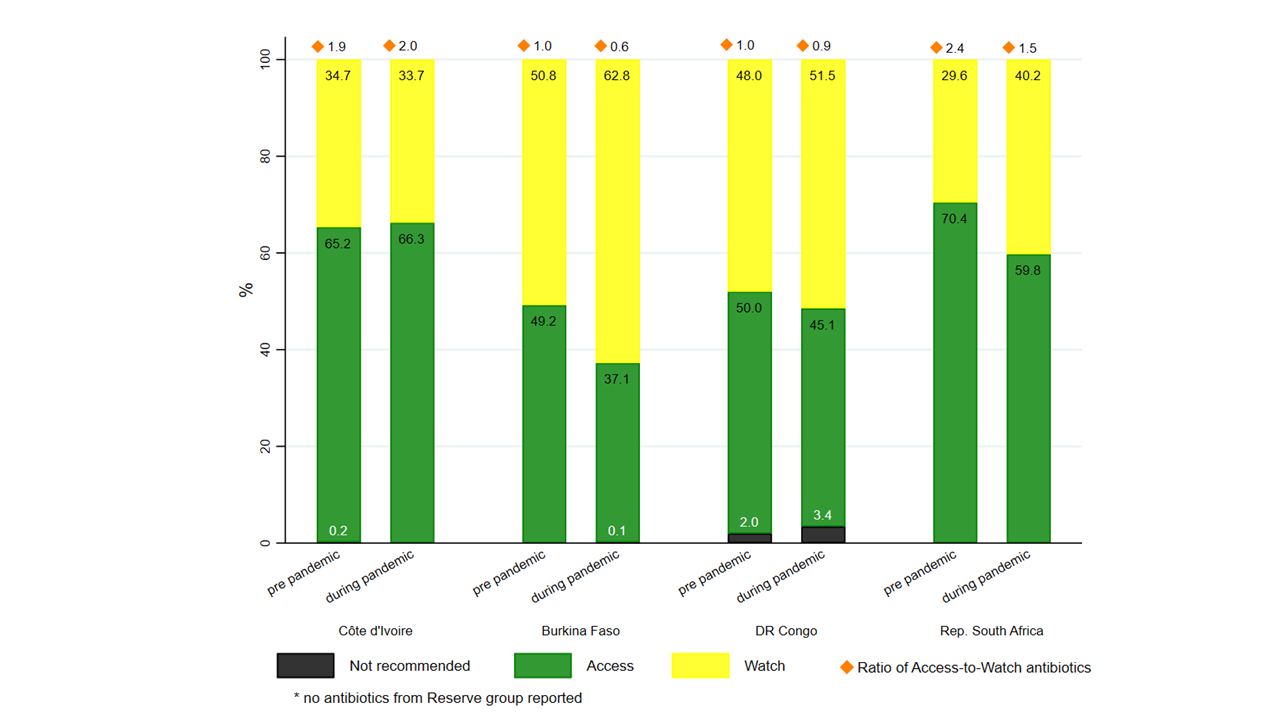


Legend: DR Congo: Democratic Republic of the Congo, Rep. South Africa: Republic of South Africa
