# Supplementary material for: Reported antibiotic use among patients in the multicenter ANDEMIA infectious diseases surveillance study in sub-saharan Africa
Source: Antimicrob Resist Infect Control. 2024 Jan 25;13:9. doi: 10.1186/s13756-024-01365-w (PMC10809765; doi:10.1186/s13756-024-01365-w)
Supplement: Supplementary file 10 — Additional file 10. Figure on total reported antibiotics regardless of date of last dose among ANDEMIA total as well as by country (.pdf). [file 13756_2024_1365_MOESM10_ESM.pdf]

Additional file 10

Figure: Total reported antibiotics regardless of date of last dose among ANDEMIA total as well as by country

Total (n=10,306)	CIV (n= 925)	BF (n= 3,027)	DRC (n= 2,737)	RSA (=3,617)
Ceftriaxone (30.5%)	Amoxicillin (26.8%)	Ceftriaxone (46.6%)	Ceftriaxone (27.3%)	Ceftriaxone (23.2%)
Amoxicillin (14.9%)	Ceftriaxone (16.0%)	Amoxicillin/Clav. (21.3%)	Amoxicillin (19.4%)	Ampicillin (20.4%)
Amoxicillin/Clav. (10.8%)	Amoxicillin/Clav. (15.2%)	Amoxicillin (9.9%)	Metronidazole (12.2%)	Amoxicillin (12.7%)
Ampicillin (8.2%)	Metronidazole (14.4%)	Metronidazole (6.6%)	Gentamicin (6.9%)	Gentamicin (12.5%)
Metronidazole (8.1%)	Gentamicin (7.4%)	Gentamicin (3.7%)	Ciprofloxacin (6.9%)	Sulf./Trim. (8.1%)
Other (27.5%)	Other (20.2%)	Other (11.9%)	Other (27.3%)	Other (23.2%)

Legend: CIV: Côte d'Ivoire; BF: Burkina Faso; DRC: Democratic Republic of the Congo; RSA: Republic of South Africa. Access antibiotics are colored in green and Watch antibiotics are colored in yellow, if not under top five reported antibiotic, grouped as "other". Sulf./Trim. : Sulfamethoxazole/Trimethoprim; Amoxicillin/Clav.: Amoxicillin/clavulanic acid
