# Supplementary material for: Reported antibiotic use among patients in the multicenter ANDEMIA infectious diseases surveillance study in sub-saharan Africa
Source: Antimicrob Resist Infect Control. 2024 Jan 25;13:9. doi: 10.1186/s13756-024-01365-w (PMC10809765; doi:10.1186/s13756-024-01365-w)
Supplement: Supplementary file 11 — Additional file 11. Table on the number of different antibiotic substances reported in the ANDEMIA study by country and by location (.pdf). [file 13756_2024_1365_MOESM11_ESM.pdf]

Additional file 11

Table: Number of different antibiotic substances reported in the ANDEMIA study by country and by location.

Country	Location	Number of different antibiotics reported	parenteral	oral	parenteral/oral/other
CIV	Urban	21	5	5	11
	Rural	14	2	4	8
BF	Urban	20	7	3	10
	Rural	14	4	1	9
DRC	Urban	37	12	10	15
	Rural	14	4	2	8
RSA	Urban	25	9	4	12
	Rural	17	6	2	9

Legend: CIV: Côte d'Ivoire; BF: Burkina Faso; DRC: Democratic Republic of the Congo; RSA: Republic of South Africa.
